# Financial literacy among young college students: Advancements and future directions 

**DOI:** 10.12688/f1000research.159085.3

**Published:** 2025-08-28

**Authors:** Paula Andrea Rodríguez-Correa, Sebastián Arias García, María Camila Bermeo-Giraldo, Alejandro Valencia-Arias, Ezequiel Martínez Rojas, Edward Florencio Aurora Vigo, Ada Gallegos

**Affiliations:** 1 Centro de Investigaciones, Fundacion Escuela Colombiana de Mercadotecnia, Medellín, Antioquia, 050012, Colombia; 2 Departamento de Ciencias Administrativas, Instituto Tecnologico Metropolitano, Medellín, Antioquia, 050034, Colombia; 3 Escuela de Ingeniería Industrial, Universidad Senor de Sipan, Chiclayo, Lambayeque, 14001, Peru; 4 Vicerrectoría de Investigación e Innovación, Universidad Arturo Prat, Iquique, Tarapacá Region, 1110939, Chile; 5 Escuela de Ing. Agroindustrial y Comercio E, Universidad Senor de Sipan, Chiclayo, Lambayeque, 14001, Peru; 6 Departamento Académico de Ciencias jurídicas y Políticas, Universidad Nacional Mayor de San Marcos, Lima, 15081, Peru

**Keywords:** Financial literacy, College students, Finance skills, Financial Behavior, PRISMA-2020

## Abstract

Financial literacy is an essential life skill for young adults, particularly college students facing increasing financial responsibilities. This study conducted a systematic literature review following the PRISMA methodology, analyzing 44 peer-reviewed studies to identify the most prevalent dimensions of financial literacy among college students. The results show that while research focuses primarily on broad categories such as financial knowledge and behavior, more specific subtopics, such as budgeting, credit card use, student loans, and digital financial tools, are unevenly addressed or under-explored. This article contributes by mapping these subtopics, identifying research gaps, and proposing a structured research agenda that prioritizes financial behaviors relevant to the youth context. The findings provide practical insights for educators, curriculum developers, and policymakers seeking to design financial education strategies targeting the college student population.

## 1. Introduction

Nowadays, the adequate management of personal finances is important. Poor financial decision-making by consumers when dealing with financial products and services, such as savings and checking accounts, credit cards, and mortgage loans, indicates that there is a need to improve financial education ( Goyal K. & Satish K., 2021). For low-income people, there are products without a minimum deposit, which significantly expands the range of financial products available to audiences that previously had no accessibility ( Garg N. & Shveta S., 2018).

Different varieties of financial offers generate greater autonomy for people to make financial decisions, such as saving or investing, which implies greater responsibility ( Stolper O. & Andreas W., 2017). In this sense, access to credit, the digitization of banking, increased longevity and the outlook for retirement demand financial education for adequate decision-making in matters of daily expenses, emergency funds, educational funds, mortgage funds and retirement ( Goyal K. & Satish K., 2021).

Young people represent one of the populations most vulnerable to financial abuse, as the decisions they make can affect their lives over a long period. It is imperative for young people to focus on understanding the world of finance to adequately choose and manage financial products ( Garg N. & Shveta S., 2018). Experts agree that financial knowledge is related to better financial behavior to take effective measures in the current and future management of money ( Oseifuah, 2010). In the context of this study, the term ‘youth’ refers specifically to college students, generally in the 18-24 age range. This population group is relevant because they are in a transitional stage toward economic independence and making key financial decisions that affect their future.

Financial literacy is one of the most crucial skills required of people in the 21st century. Knowledge and confidence in economic participation are part of the financial literacy that young people need to acquire to engage in economic markets without great risk ( Shahid, 2022). Key components and methods of effective financial education for young people should be identified to guarantee the long-term fiscal well-being of young people, their families and economic development in general ( Totenhagen et al., 2015). In the long term, in general, low savings rates, increased debt and lack of savings for retirement can occur, which suggests that financial education is necessary to increase and strengthen knowledge, skills and changes in financial behavior among the young population ( Oseifuah, 2010).

Although there is growing recognition of the importance of financial education for young people, its effective implementation remains a challenge in several countries, especially in emerging economies, due to limitations in resources, public policies and educational strategies ( Samy et al., 2008). Motivated by the above, in this study, research on financial literacy among young people is reviewed to identify the most important factors related to financial literacy among young college students.

### 1.1 Financial literacy

The definition of financial literacy is broad and is closely linked to financial education. Its importance has been emphasized for many years based on the need to obtain basic knowledge about the nature of money ( Goyal K. & Satish K., 2021). Across time, the study of financial education and how people learn about relevant topics such as credit, circulation of money has been promoted ( Garg N. & Shveta S., 2018). Financial literacy refers to the ability of people to make adequate decisions regarding their financial life based on judgements created from training in topics about the nature and circulation of money, credit, savings for education, retirement, accidents, pensions, among others ( Taylor S. & Suzanne W., 2011).

According to Lusardi and Mitchell (2023) financial literacy means people’s knowledge of and ability to use fundamental financial concepts in their economic decision-making. Another definition explains financial literacy as “the ability to use knowledge and skills to manage financial resources effectively for a lifetime of financial well-being” ( Knoll and Houts, 2012, p. 383). Under this approach, Blanco et al. (2024) highlight that the social determinants of financial knowledge, behavior, and well-being are also necessary to address racial/ethnic and gender disparities in finance.

Therefore, some authors highlight the transition from the term financial literacy to other more inclusive terms, such as financial capability ( Xiao et al., 2022) or financial well-being, including skills to apply knowledge in everyday life ( Bartholomae and Fox, 2021). Consistent with these definitions, this study analyzes financial literacy through the lens of financial knowledge, behavior, and attitudes, which are key components to understanding how college students manage their financial lives.

According to Oseifuah (2010), some key elements of financial education skills and knowledge are related to mathematical knowledge, the nature and forms of money, attitudes towards spending and saving, awareness of risks associated with financial products and the ability to make responsible and conscious decisions.

The importance of financial literacy lies in greater financial inclusion, improvements in the economy and the strengthening of the financial sector. People, for example, do not make adequate financial plans to cover their expenses, acquire financial products and services that fail to meet their actual needs, and even become victims of exploitative practices and scams ( Aren S. & Sibel D., 2014). Financial literacy is a life skill, a requirement for citizenship and a critical intellectual competence that is essential for young people (such as university students) to acquire to perfect critical thinking, judgement, and other skills of a responsible citizen ( Kezar & Hannah, 2010).

### 1.2 Financial literacy among college students

For several years, the need to include financial education has long been recognized as essential within college curricula. Before entering the labour market, students should master personal finance skills and have acquired training in responsible attitudes and sound financial behavior within their academic curriculum ( Kezar & Hannah, 2010; Potrich et al., 2016). A greater inclusion of financial education in the academic programs of universities and higher education institutions would help students learn to properly manage their finances and improve their financial well-being, especially under the environmental and technological influences of the modern world ( Ergün, 2018).

Some studies on this topic have focused on emerging economies. For example, Lantara I. Wayan N. & Ni K. R. K. (2015) investigates the level of financial education among undergraduate and graduate students in Indonesia. They found that male students, students with economics and business majors, and those with higher incomes and more work experience have higher rates of financial literacy. Other studies have examined students’ knowledge of savings and spending, banking, risks and insurance, investments, and general financial knowledge ( Sarigül, 2014). Other studies have examined models that integrate the financial knowledge, behaviors, and attitudes of university students in Brazil, finding that financial knowledge and attitude have positive impacts on the financial behavior of students ( Potrich et al., 2016). The level of financial education among university students in developed countries has also been addressed. Ergün (2018) found that male students, business students, and doctoral students who live in a rental house, students whose parents have a high level of income, students who receive advice on financial matters from their friends who have previously taken financial courses, and students who obtain financial information from college education have more knowledge about personal finances. Based on the above, this research aims to identify the most relevant issues related to financial literacy among young university students.

In addition, it is important to note that financial literacy among university students varies considerably by region and country. Access to financial education programs, the quality of the curriculum and institutional support differ by context. In some countries, formal financial education is integrated into university programs, while in others it is absent or minimal, so socioeconomic conditions, gender and cultural values also influence how financial knowledge is acquired and applied Ahmad N. L. et al. (2021). Thus, comparative and context-specific studies are essential to fully understand the financial literacy landscape of this population.

## 2. Methods

In accordance with the purpose of the research, it is proposed to conduct an exploratory study based on secondary sources of information. This approach involves conducting a systematic literature review, following the methodology defended by Rejeb A. et al. (2022). This methodology is characterized by its ability to provide a quantitative evaluation of the current state of the scientific corpus around a specific topic. Furthermore, it allows the projection of possible future research directions, thus offering perspectives on emerging trends. To further strengthen this comprehensive literature review, the criteria outlined in the PRISMA statement are adopted. This statement, as detailed in Page M. J. et al. (2022), establishes rigorous and effective guidelines for conducting literature reviews.

### 2.1 Search strategy

The search strategy is designed to take into account the nature of international databases, which require searches in English, as well as the previously detailed inclusion criteria. In this sense, the following specialized search strings are established:

For the Scopus database: (TITLE (“Financ* literacy” OR “Financ* literate” OR “Financ* education”) AND TITLE (“University student*” OR “College student*” OR students))

For the Web of Science database: (TI= (“Financ* literacy” OR “Financ* literacy” OR “Financ* education”) AND TI= (“University student*” OR “College student*” OR students))

### 2.2 Inclusion and exclusion criteria

As eligibility criteria, inclusion and exclusion criteria are established to select the most relevant documents according to the objective of the review. Three rounds of review were carried out by all the researchers: first, a review of the title, abstract, and keywords was conducted to exclude those records that were not in line with studies on financial literacy among university students. In a second review, those documents that did not include variables measuring financial literacy were excluded. Finally, the documents were scored from 1 to 3 based on their relevance to the research objective and the quality of their content (see Table 1). In this way, the relevance of the selected documents was assessed in alignment with the research objective.

**Table 1.** Quality evaluation checklist.

Sr	Ask
1	Is the methodological design used well specified?
2	Is the method of analysis used well specified?
3	Is the type of population analyzed well specified?
4	Are variables for measuring financial literacy included?
5	Is there a statistical method to measure the variables?

Note: Author's calculations based on Herrera-Franco G. et al. (2020).

As part of the exclusion criteria, it was decided not to include documents that focus on populations of primary or secondary school students. Instead, we chose to include those that specifically address financial literacy among higher education students. In addition, articles that lack variables or measures of financial literacy that are analyzed using statistical methods were excluded.

With this approach, a classification system was established that assigns a score of 1 to documents that cover financial literacy factors, even if their analysis is qualitative. The Documents that perform a quantitative descriptive analysis of the financial literacy variables receive a score of 2. Finally, the documents that conduct a correlational study of the variables related to financial literacy are given a score of 3. Therefore, only documents with a score of 3 were included, while those scoring 1 or 2 were considered not relevant.

### 2.3 Sources of information

Having established the previous considerations, the two main databases currently used to index quality scientific literature, such as Scopus and Web of Science, are defined as sources of scientific information ( Caputo & Kargina, 2022). In order to carry out bibliometric analyses and literature reviews, researchers usually use these two databases, either individually or jointly, since they are the largest and have the greatest coverage of publications ( Echchakoui, 2020).

### 2.4 Risk of bias assessment

The systematic review of the financial literacy literature requires a careful process of assessing the risk of bias of the selected studies. A detailed description of the methods and tools used is essential for this assessment. In the present review, the risk of bias was assessed using an automated tool developed in Microsoft Excel. This process was carried out jointly by all the authors, including data collection, thus ensuring consistency and uniformity in the procedure. This methodology guarantees the quality and integrity of the results, as it allows a standardized and collaborative assessment of the included studies.

### 2.5 Selection process

**Figure 1.**
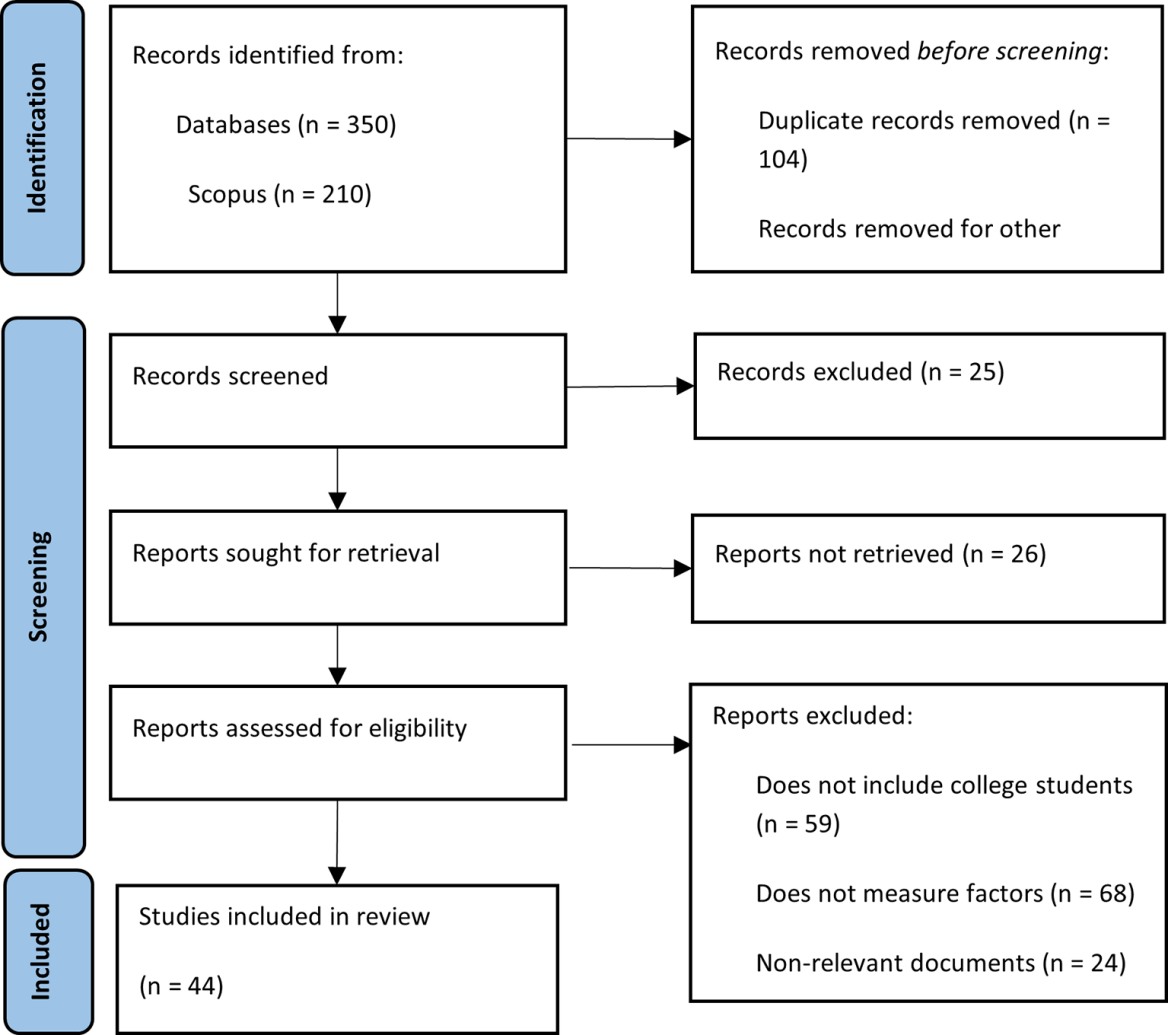
PRISMA methodological summary. Source: Own elaboration based on publications retrieved from Scopus and Web of Science.

The implementation of the search strategy in the selected databases yielded a total of 350 records related to the topic of financial literacy among university students. Of these, 210 came from Scopus and 140 from Web of Science. After removing duplicate records, a total of 246 unique documents remained. Books, lecture notes, editorials, review articles, and documents in languages other than English or Spanish were excluded, along with those that were inaccessible. After applying the predefined inclusion and exclusion criteria, 46 documents were selected that aligned with the central theme and scope of this research. This process is illustrated in Figure 1.

## 3. Results

The analysis of the results is based on the 44 selected studies listed in Table 2. These studies were published within the time window from 2003 to 2023, which served as the temporal frame for the bibliographic search and data analysis. Table 2 shows the type of document, mostly scientific articles, the objective of the study, the statistical analysis method used, and the country in which the study was applied. The time window is from 2003 to 2023. It is evident that the majority 34% use regression analysis to measure the relationship between variables from a predictive approach, followed by factor analysis (20%) and Structural Equation Modeling (SEM) (18%). In terms of countries studied, the participation of Indonesia stands out among the countries studied, followed by Malaysia, the United States, and Turkey.

**Figure 2.**
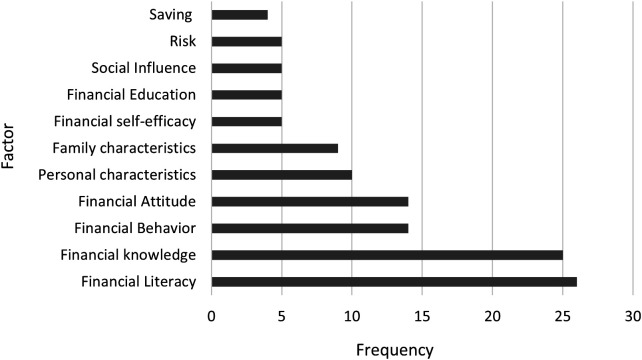
Most recurring financial literacy variables.

A careful selection was made of the most recurrent factors evaluated in the assessment of financial literacy among university students. As expected, financial literacy turned out to be the most evaluated construct in the selected studies (see Figure 2). However, a more detailed analysis revealed that this term is used with diverse scopes, from cognitive knowledge to behavioral practices and attitudinal factors. This reinforces the need for more precise and multidimensional frameworks in future research.

This concept is commonly defined as the ability to understand and manage personal finances, including budgeting, saving, using credit responsibly, planning for the future, and making informed financial decisions ( Khalisharani et al., 2022). In this context, financial literacy involves the understanding, monitoring, and effective use of financial resources with the goal of promoting the well-being and ensuring the economic stability of individuals ( Ajayi T. A. et al., 2022).

Within this panorama, studies such as that of Ahmad N. L. et al. (2021) explore financial education in the context of accounting students, finding that the financial education and financial behavior of accounting students were at a moderate level, although with a high level of business motivation. Researchers have also examined how financial self-efficacy how financial self-efficacy, which is an individual’s belief in managing finances, using financial services, and beliefs about their personal abilities, helps students achieve important financial goals ( Kartawinata B. et al., 2021).

Financial knowledge also appeared frequently in the studies analyzed. However, this prevalence should be interpreted with caution, as many researchers use financial knowledge and financial literacy interchangeably, often without explicitly defining their conceptual boundaries. Some authors define financial knowledge as students’ familiarity with some financial terms and concepts that are needed to function in society on a daily basis ( Aydin A. E. & Selcuk E. A., 2019). Other studies have found that students with low financial knowledge can engage in behaviors that, in turn, can have academic implications. For example, students may misuse financial products such as credit cards, creating uncontrollable debt while building an inadequate credit history ( Starobin et al., 2013). In line with this, Xiao et al. (2014) found that early exposure to financial literacy education is positively associated with more responsible financial behavior in later stages of college life. This reinforces the notion that building financial knowledge early can have long-lasting effects on students’ financial decision-making and outcomes.

Third, financial behavior was assessed in 14 studies. This topic, which examines how students manage their finances has been approached from multiple perspectives, including longitudinal studies aimed at evaluating the relationship between competency-based financial planning curricula and students’ financial knowledge, as seen in studies by Danes S. M. et al. (2016), and the impacts on student behavior and financial patterns according to attitudes and learning acquired in personal finance courses, as in the study by Johan I. Rowlingson K. & Appleyard L. (2021). Therefore, these studies have developed models to explain financial behaviors among university students, such as the Theory of Family Financial Socialization and the Theory of Planned Behavior.

Additionally, Henager et al. (2021) examined how different funding strategies used to pay for college impact students’ financial satisfaction. Their findings emphasize that the way students finance their education—such as through loans, scholarships, or parental support—can significantly influence their perceived financial well-being and overall financial behavior, reinforcing the importance of context in understanding student financial outcomes.

Financial attitude has also been evaluated in 14 studies and is related to financial decision making through a process where students assess whether a financial decision is appropriate or necessary ( Gilenko E. & Chernova A., 2021). In this way, it refers to an individual’s state of mind, opinion, and judgment regarding their finances, and has therefore been linked to financial behavior and financial literacy ( Khalisharani et al., 2022).

Furthermore, it is evident that among the most measured variables are personal characteristics, e.g., age, gender, ethnicity, race, and nationality. Likewise, family characteristics have been an important measure of financial literacy, highlighting parental education as part of an analysis related to the socio-economic environment of students, based on the assumption that elements of household culture influence financial decisions in financial decisions ( Cordero & Pedraja, 2019).

To improve the interpretation of the findings and respond to the need for a more nuanced understanding of financial literacy among college students, the variables identified in the 44 selected studies were then grouped into six thematic subcategories. This thematic organization was based on an inductive classification of recurring variables reported in the literature, as recorded in the analysis matrix.

### 3.1 Core financial knowledge

Most studies focus on students’ understanding of basic financial concepts ( Beal & Delpachitra, 2003), such as interest rates, inflation, savings mechanisms, and credit ( Tejada-Peña et al., 2023). However, deeper concepts such as compound interest, long-term investing, and retirement planning are addressed in fewer publications, despite their relevance to early financial habits.

Related studies have argued that most college students calculate compound interest and, consequently, tend to make mistakes when interpreting financial information associated with the concept of interest rates ( Moreno-García, 2024; Moreno-García et al., 2017). In that sense, financial knowledge requires understanding the calculation of interest on loans (simple interest concept) and interest on deposits (compound interest concept), as well as awareness of investments ( Channak et al., 2022). In that sense, intervention in curricula, textbooks, and courses in microeconomics and macroeconomics becomes necessary at both undergraduate and graduate levels to address these topics, as well as saving for retirement, budgeting, investing for the long term, and disposing of their assets in old age ( Lusardi and Mitchell, 2023).

### 3.2 Budgeting and spending habits

A recurring theme in the literature is the ability of students to create and follow personal budgets. Budgeting is considered a method to improve effective money management; however, globally, students are the least interested in this process, which evidences the need to intervene in financial education within universities ( Phung, 2023). Budgeting is discussed about income management, discretionary spending, and financial stress. Income generally comes from salaries, business, and investments, and in the case of some university students, from scholarships or family support, and the literature has shown that financial literacy and income have a positive and significant effect on personal financial management ( Pamella, 2022). In turn, the increase in income associated with entering the full-time labor market requires increasing some money management behaviors, such as discretionary spending ( Sinnewe and Nicholson, 2023).

For their part, Johan et al. (2021) have highlighted that, as college students begin living independently, they face new responsibilities for managing their finances, including budgeting, managing income and expenses, and paying bills. Another concern has to do with awareness of investment scams among college students. In the study by Mohd Padil et al. (2022) they found that having adequate budgeting skills can significantly improve awareness of investment scams among students. However, few studies address how students adapt their budgets to irregular income sources (e.g., part-time jobs or scholarships).

### 3.3 Credit and debt management

While several studies address the use of credit cards and student loans, many do not analyze their psychological and behavioral effects ( Zachary Finney and Finney, 2018). Previous studies show that the relationship between financial literacy and risky credit behavior intensifies when college students’ financial stress levels are high ( Liu and Zhang, 2021). Andrews (2021) mentions that the explosion of access to consumer credit from the 1980s through the 2000s, college students are using credit cards, often to cover shortfalls in their budgets while trying to pay for college.

The literature reveals that poor debt management is common, and few students understand credit limits, interest accrual, and repayment obligations ( Chen et al., 2023). In that sense, an interest has arisen in recent years in the financial well-being line of research driven by the onset of the “student debt crisis” and how factors such as financial behaviors, individual and personal characteristics, family relationships, and formal socialization processes influence this phenomenon ( Bartholomae and Fox, 2021).

### 3.4 Financial technology (FinTech) and digital tools

Recent research links financial literacy to digital platforms ( Yin Yin et al., 2022), such as budgeting apps ( Kamarudeen and Vijayalakshm, 2023), digital banking ( Respati et al., 2023), and online financial education ( Agasisti et al., 2023). Previous studies have explored how financial technology acts as an enabler of financial inclusion and financial literacy, which requires understanding how financial literacy drives behavior change, capacity building, and economic resilience ( Croitoru et al., 2025). These technologies are especially relevant for digitally literate students, but the literature reveals a gap in assessing their actual effectiveness in improving literacy ( Bermeo-Giraldo et al., 2023).

Previous studies have argued that while easily accessible fintech products are on the rise for consumers, **a** lack of basic financial literacy (FTL) may prevent them from taking full advantage of their benefits ( Khan et al., 2023). Thus, as highlighted by AlSuwaidi and Mertzanis (2024) higher levels of financial literacy in a population could **catalyze** fintech market expansion. Thus, this is a potential research opportunity in the context of financial literacy among university students.

### 3.5 Financial attitudes and self-efficacy

Another important issue surrounding financial literacy has to do with financial self-efficacy, i.e., the college student’s confidence in his or her ability to acquire information to make effective financial decisions ( Lone and Bhat, 2022). Another important factor is financial attitude, as previous studies such as Mustafa et al. (2023) showed that financial attitude and financial literacy significantly influence retirement planning, an essential factor in ensuring that individuals have enough money to live their desired lifestyle in retirement.

Studies explore students’ confidence in money management ( Tejada-Peña et al., 2023), their financial goals ( Lone and Bhat, 2022), and their emotional responses to financial decisions ( Rosales-Pérez et al., 2021). Financial self-efficacy emerges as a moderating factor between knowledge and behavior ( Amagir et al., 2022), although instruments to measure it vary considerably.

### 3.6 Socio-demographic and cultural variables

In recent years, numerous articles have evaluated sociodemographic and cultural factors such as the impact of gender, parental education, income, and field of study on financial literacy ( Hanson, 2022; Sahabuddin and Hadianto, 2023). However, in the literature, cultural and class determinants, such as social norms around savings or perceptions of indebtedness, are often overlooked ( Yanto et al., 2021). For as explained by Blanco et al. (2024) the financial information needs of women and racial/ethnic minorities can help reduce racial/ethnic and gender gaps in financial wellbeing. In addition, young people need to think about the actual or planned implementation of various retirement strategies, including private pension funds, asset investment, government subsidies, and family support ( García Mata, 2021). This suggests the need for more context-specific research.

### 3.7 Research gaps

The systematic review of financial literacy led to the development of Table 3, which summarizes the main gaps identified in the existing literature. These gaps represent underexplored areas or issues that require further investigation and are crucial for guiding future lines of inquiry. The purpose of highlighting these gaps is to offer a framework that informs future studies and ensures that the most relevant and pressing issues are addressed, thereby enriching the academic understanding of financial literacy. Scholars working in this field are encouraged to review and consider these gaps when designing upcoming research projects.

**Table 3.** Research gaps. Author's calculations based on Scopus and Web of Science.

Category	Identified gaps	Justification	Questions for future researchers
Thematic Gaps	Socioeconomic variables	Variables such as parental education have been considered, but the relationship between social class and financial literacy has not been explored in depth ( Cordero J. M. & Pedraja F., 2019).	How does social class affect financial literacy?
	Financial Literacy and Technology	The relationship between financial literacy and financial technology use is unknown.	How is fintech impacting the financial literacy of college students?
Geographic Gaps	Emerging Markets	Research in specific countries is highlighted, but there is a lack of studies in other regions, such as emerging markets.	How does financial literacy compare among college students in emerging markets?
	Eastern Europe	Although Western countries are well represented, Eastern Europe may be underrepresented.	What is the state of financial literacy in Eastern European countries?
Interdisciplinary Gaps	Psychological Perspective	While financial behavior has been studied, a deeper understanding from psychology could be beneficial.	What are the major psychobehavioral theories that explain the factors that determine financial literacy?
	Sociocultural approach	The home culture and socioeconomic environment are relevant ( Cordero J. M. & Pedraja F., 2019), but an anthropological or sociological perspective could provide new analytical perspectives.	How do cultural practices and traditions affect financial literacy?
Temporary gaps	Updates after 2023	The review must be kept up to date to reflect changes and emerging trends.	What are the emerging trends in financial literacy beyond 2023?

## 4. Discussion

This study proposes a research agenda that identifies the most recurrent research topics, as well as the years of greatest relevance of the keywords. In this way, it is possible to define those topics that have already lost relevance and those expected to gain relevance in upcoming research. The analysis shows that the most addressed topic has been credit.

**Figure 3.**
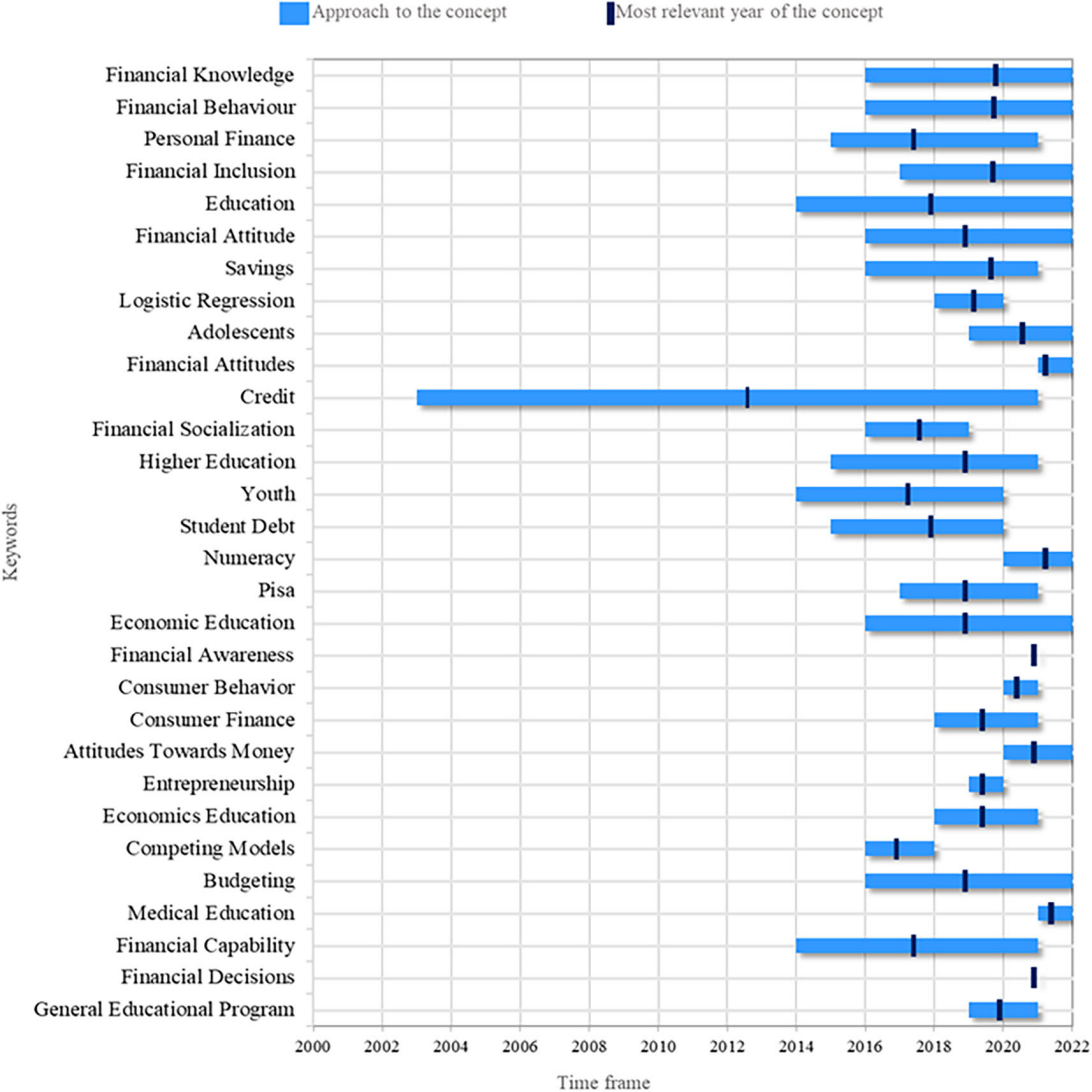
Research agenda. Source: Own elaboration based on publications retrieved from Scopus and Web of Science.

In Figure 3, it is possible to illustrates the topics that are currently relevant and tend to increase in the coming years. Among these topics, Financial Knowledge stands out, which is closely related to the research object of this study, as it is a knowledge that allows people to understand information related to personal finances and business. Financial Behavior is also a topic of great interest at present and in the future by researchers, related to people’s reasoning that affects financial decision-making. On the other hand, recent studies have also addressed Financial Inclusion, which refers to access to useful and accessible financial products and services according to each person’s needs.

There is also sustained interest in the topic of Budgeting for budget management, whether personal or business. Adolescents are a target audience for financial literacy re-search, as they are at a crucial age to learn about personal finances and thus influence better financial decisions they may make in the future. Therefore, it is another topic with a promising future in future research. On the other hand, from the theory of human behavior, the study of variables such as Attitudes Towards Money has intensified in order to identify a young person’s favorable or unfavorable position towards money. To this topic, the theme of Financial Attitude is also added, referring to someone’s willingness to behave towards financial literacy.

In this way, new research questions may arise from research gaps that can be ad-dressed in future studies. What improvement strategies can be implemented in universities in emerging economies to strengthen the financial literacy of university students? How can innovative behavior influence financial decisions in adolescents? What strategies can be implemented to improve the personal finances of young people in developing economies? How can financial inclusion influence better financial decision-making among university students?

The results of this study allowed the authors to meet the objective of identifying the most relevant issues pertaining to financial literacy among young university students, providing a structured guide for future research. With the development of the methodology based on PRISMA, a more detailed and structured process for retrieving publications was developed ( Barquero, 2022). This systematic review collected and processed a total of 44 documents that focus on financial education and college students. Unlike previous studies that adopted a broader focus on financial well-being ( Nguyen S. M., 2022), reviewing the issue in a period from 2002 to 2022 ( Ansari et al., 2022), from reviews of learning methodologies on financial education ( Hussein J. K. & Hussein K. S., 2022), this review adopted a more specific perspective regarding a population that is very important for consumption and economic development, i.e., young university students.

The results indicated that financial literacy among young university students is a growing field. This finding is consistent with those reported by Méndez S. et al. (2022), who analyzed the current state of the literature in Latin America and the Caribbean, and by Shelly et al. (2022), who addressed the personal finances of the general population. These two investigations indicate progress and a growing global interest in the topic.

On the other hand, it should be recognized that there are some critical issues with the term *financial literacy*. Firstly, the concept has evolved, giving way to broader notions such as *financial capability* and *financial well-being*. In this sense, financial literacy has traditionally been related to the knowledge and understanding of financial concepts, products, and services. More recently, financial capability has come to include the skills and behaviors needed to apply this knowledge to financial decision-making. Financial well-being focuses on the results of financial behavior, aiming to help individuals feel secure and in control of their finances now and in the future. Recent studies indicate that the field of financial literacy is shifting toward these comprehensive frameworks, seeking to reflect that individuals not only possess financial knowledge but also apply it effectively and how they feel about decisions about their financial lives ( Lone & Bhat, 2022; Kamble et al., 2024).

In that sense, although this review study focuses specifically on financial literacy given its importance in the academic literature and the relevance that still persists in higher education, mainly in emerging economies, the integration of concepts such as financial wellbeing and financial capability, which are broader, could enrich future research. Incorporating these perspectives would allow for a more comprehensive understanding of the financial realities faced by college students and, in turn, could support the development of more effective educational interventions and public policies aimed at promoting the long-term financial health of young people ( Park et al., 2024; Jaffar et al., 2025).

### 4.1 Limitations

In terms of limitations, it is important to highlight that the search strategy limits the scope of the study. The time frame (2003-2023) covered two decades of research; thus, the scope of the search was limited to the terms Finance*, literacy, education, University student*, College student, and students. Therefore, it is possible that studies that addressed the topic but did not explicitly mention the search terms in their titles, may have been excluded. Thus, terms such as “university” and “College” were not included independently in the original search equation, nor was the abstract considered as a search field. In that sense, such a methodological decision may have limited the completeness of the results.

This bibliometric study, although comprehensive and based on the PRISMA-2020 methodology, has some limitations that must be recognized. First, the study is limited to the Scopus and Web of Science databases, excluding other relevant academic databases that may contain relevant research on financial literacy. This limitation could lead to a biased view of the field of study. In addition, the reliance on specific tools such as Microsoft Excel® ( Excel, 2007) and VOSviewer® ( Van Eck and Waltman, 2010) which constrain the bibliometric analysis to the functionalities offered by these platforms. While these tools are widely recognized for their effectiveness, other software or analytical techniques may offer complementary perspectives or alternative methods of interpretation. Finally, the study’s focus on bibliometric indicators related to quantity, quality, and structure—although relevant—may have overlooked other important metrics that could provide a more nuanced understanding of the evolution and trends within the financial literacy literature.

### 4.2 Practical implications

Regarding the practical implications, this systematic review complemented by a bibliometric analysis aimed to offer a more comprehensive view of the dynamics of the academic and scientific production related to financial education. Therefore, this research provides inputs for policymakers, regulators, and researchers to understand the characteristics of financial education and youth behavior and to identify research fields with potential. Likewise, the findings of this research contribute to consolidating the literature in the field of financial literacy and provide areas of interest for other authors and professionals to carry out future research activities.

The analysis of the literature on financial literacy revealed that quantitative methods predominate; therefore, more qualitative studies should be developed to provide a deep understanding of the behavior of individuals with regard to managing their finances in different contexts.

The findings indicate that future studies should focus on measuring financial illiteracy in various economic contexts because the results of the analysis suggest that financial illiteracy is a phenomenon not only in developing countries but also in advanced economies. Studies that measure the levels of financial education in different population generations, such as Millennials and Centennials, in which financial behavior and knowledge can be influenced by technology, should also be conducted.

Other future studies could analyze emerging economies, with longitudinal studies that allow comparisons of the evolution and new needs demanded by financial markets; the latter will determine the training content required in the financial field.

The systematic review of the literature on financial literacy, through an exhaustive synthesis and analysis of scientific works in the field, has profound practical implications in various sectors and dimensions. One of these is education. By identifying dominant trends and themes in the literature, education systems can adapt and update their curricula to incorporate modern concepts and pedagogical methods in the teaching of financial literacy. This is particularly relevant at the secondary and tertiary levels, where young people begin to have their first experiences with personal financial management.

From a public policy perspective, a robust systematic review of financial education can inform decisions about the design and implementation of national programs aimed at improving the financial health of the population. For example, by identifying geographic areas or demographic groups that are underrepresented in the literature, policymakers can target resources and efforts to these populations to ensure that they are not left behind in terms of financial skills and knowledge.

Another practical implication of this type of study is for the financial sector itself. Financial institutions such as banks, credit unions and others can benefit from a deeper understanding of trends and gaps in financial literacy. This understanding can help them to design products, services and marketing campaigns that are most appropriate and effective for their customer base. In addition, by identifying areas where financial literacy is low, institutions can develop education and training programs targeted at their customers.

Finally, in the area of technological innovation, understanding the literature on financial literacy through systematic reviews can guide the development of digital tools and platforms aimed at improving financial health. FinTechs and technology start-ups can identify opportunities to address unmet needs or develop solutions that are better adapted to emerging trends in financial behavior, particularly in the context of rapid digitization and technological change.

## 5. Conclusions

In this study, the research on financial literacy among young university students was reviewed, and the most relevant issues around this phenomenon were identified. Two themes remain prevalent and are the most frequently discussed in the literature on financial literacy among university students: “ *Financial Behavior*” and “ *Financial Knowledge*”.

Keyword mapping identified five thematic groups that are currently active in the literature on financial literacy among college students: (1) gender studies on the management of personal finances and savings; (2) determinants of financial education for self-efficacy and financial inclusion; (3) behavior and social awareness of financial training by students; (4) knowledge of financial well-being and basic fundamentals of financial literacy; and (5) use and adoption of financial services by university students.

Recently, financial education and literacy have increased, occupying a leading role in economic development and individual well-being, thus making it imperative to investigate the level of financial education among different segments of society. It is essential to assess people’s financial knowledge, and the ability to correctly use financial instruments and methods must be promoted. Based on identified financial deficiencies, appropriate individualized training programs can be developed.

This research has some limitations that may be characteristic of the literature review methodology, such as the fact that only the Scopus and Web of Science databases were used to obtain publications for the analysis and calculation of bibliometric indicators. Therefore, other researchers can include other databases to complement the study. Additionally, the keywords used for the search equation should be reconsidered because they may not be sufficient, i.e., the search could be expanded to include new keywords related to skills and financial management. This could provide more relevant information for making more accurate financial decisions. Finally, this study may have excluded high-quality research published in languages other than English.

Another important limitation lies in the scope of the search strategy. While the study followed the PRISMA 2020 guidelines and used widely accepted descriptors such as “financial literacy” and “financial education,” the search equation did not include related terms such as “financial knowledge” or “financial skills,” which are often used interchangeably in the literature. This limitation in terms of search terms prevented capturing additional relevant publications that approach the topic from slightly different conceptual perspectives. Therefore, it is recommended that future research around the topic design a broader keyword strategy to ensure even greater coverage and the inclusion of diverse definitions and concepts.

### Ethics and consent statement

No ethical approval or consent was required.

## Disclosure statement

No

## Data availability

### Underlying data

No data are associated with this article.

### Extended data

Zenodo: Financial Literacy among Young College Students: Advancements and Future Directions, https://doi.org/10.5281/zenodo.14204753 ( Rodriguez-Correa et al., 2023).

The project contains the following data:

• Database. Csv
• Figure 1
• Figure 2
• Figure 3
• Flowchart
• PRISMA checklist
•
Table 2

The repository contains the data extracted from the databases for the bibliometric analysis. It also includes the PRISMA-recommended checklist for systematic literature reviews, along with the corresponding flow diagram.

The data availability statement for this study has been duly registered and archived in the Zenodo open data repository, which is recognized for its commitment to the accessibility and preservation of scientific data. The data and materials supported by this study are publicly available under a Creative Commons Attribution 4.0 International (CC BY 4.0) license and can be accessed at the following DOI link: https://doi.org/10.5281/zenodo.14204753 ( Rodriguez-Correa et al., 2023).
