# Supplementary material for: Financial literacy among young college students: Advancements and future directions 
Source: F1000Res. 2025 Aug 28;14:113. Originally published 2025 Jan 20. [Version 3] doi: 10.12688/f1000research.159085.3 (PMC12411838; doi:10.12688/f1000research.159085.3)
Supplement: Peer review history

# Reviewer report: Reviewer response for version 3

**DOI:** 10.5256/f1000research.184466.r409697

**Published:** 2025-09-04

**Authors:** Luisa Blanco

**Affiliations:** 1 Pepperdine University, Malibu, USA

Thanks for addressing my comments.

Are the rationale for, and objectives of, the Systematic Review clearly stated?

Yes

Is the statistical analysis and its interpretation appropriate?

Partly

If this is a Living Systematic Review, is the ‘living’ method appropriate and is the search schedule clearly defined and justified? (‘Living Systematic Review’ or a variation of this term should be included in the title.)

Partly

Are sufficient details of the methods and analysis provided to allow replication by others?

Partly

Are the conclusions drawn adequately supported by the results presented in the review?

Partly

Reviewer Expertise:

Financial behavior, financial knowledge

I confirm that I have read this submission and believe that I have an appropriate level of expertise to confirm that it is of an acceptable scientific standard.

# Reviewer report: Reviewer response for version 2

**DOI:** 10.5256/f1000research.180205.r382499

**Published:** 2025-05-28

**Authors:** Terron Phillips

**Affiliations:** 1 Purdue University (Ringgold ID: 311308), West Lafayette, Indiana, USA

This is an extremely important topic that will only increase in relevance with time as millenials and Gen Zers are making more and more serious financial decisions post-college; thank you for your work researching financial literacy in formal educational environments. I appreciate the methodological approach, as it's a useful (and somewhat uncommon) and effective means to address your research question/overall study aim. I do wonder, however, about the novelty of the findings and their potential contributions to the scholarship on financial literacy in higher education specifically. There's a growing body of literature out there (you cite Aydin & Seluk, I also recommend checking out Phillips & Kiracofe, 2022 (Ref 1), Hernandez & Gibb, 2020 (Ref 2); Moreland-Capui, 2019 (Ref 3) on finanicial literacy programming and development in higher education, and the core skills and competencies typically needed by college-age students. Findings like the importance of budgeting, financial planning, social/ cultural responsive financial ed, the relationship between financial behaviors and knowledge, etc. are evident across a number of studies. What I think is needed are concrete recommendations toward designing and implementing programming at the higher ed level that addresses each of the themes highlighted in this and previous studies.

Are the rationale for, and objectives of, the Systematic Review clearly stated?

Partly

Is the statistical analysis and its interpretation appropriate?

Yes

If this is a Living Systematic Review, is the ‘living’ method appropriate and is the search schedule clearly defined and justified? (‘Living Systematic Review’ or a variation of this term should be included in the title.)

Not applicable

Are sufficient details of the methods and analysis provided to allow replication by others?

Yes

Are the conclusions drawn adequately supported by the results presented in the review?

Yes

Reviewer Expertise:

Area of expertise: Higher Education Leadership, Education Law & Policy,  Financial Literacy/wellness in higher educatiuon, culturally responsive higher education

I confirm that I have read this submission and believe that I have an appropriate level of expertise to confirm that it is of an acceptable scientific standard.

## References

[1] : Financial Literacy Programming in Higher Education: What’s There and What’s Missing. *Journal of Student Affairs Research and Practice* .2023;60(5) : 10.1080/19496591.2022.2074796 702-715 10.1080/19496591.2022.2074796

[2] : Culture, behavior and health. *Evol Med Public Health* .2020;2020(1) : 10.1093/emph/eoz036 12-13 10.1093/emph/eoz036 31976074 PMC6970345

[3] : Training for Change.2019; 10.1007/978-3-030-19208-2 10.1007/978-3-030-19208-2

# Reviewer report: Reviewer response for version 2

**DOI:** 10.5256/f1000research.180205.r382496

**Published:** 2025-05-28

**Authors:** Luisa Blanco

**Affiliations:** 1 Pepperdine University, Malibu, USA

**Major Comments**

- **Contribution of the paper** – From reading the abstract, I am not convinced that this paper offers a major contribution. I appreciate the idea behind the study, but I find the structure unconvincing. The terms *financial knowledge* and *behavior* are very broad, and the topics suggested for future research are essentially subtopics within these broad categories. I suggest organizing the literature around these various subtopics, then identifying which ones are more commonly addressed and which are underexplored. For example, when focusing on college students, it makes sense to emphasize specific financial behaviors relevant to them—such as student debt and budgeting. In contrast, topics like retirement preparedness and savings may be less applicable to this group. That said, one could argue that introducing college students to concepts like retirement savings and compound interest is crucial. If your paper identifies a gap in that area, it would offer specific insights for stakeholders.
- **Definition of financial literacy** – Please elaborate on this definition, as it is central to your study. There are several key papers that discuss this in detail. Given your focus on financial knowledge and behavior, a more comprehensive explanation is necessary. See the references below for possible sources to incorporate.
- **Financial literacy among college students** – I recommend expanding this section. The experiences of college students regarding access to financial education programs and their levels of financial knowledge likely vary across regions and countries. This section currently feels weak, especially considering it is the main focus of the paper.
- **Methods section** – I suggest reorganizing this section for better flow. For instance, instead of starting with inclusion/exclusion criteria, consider beginning with the search strategy so readers understand your starting point.
- **Search term** – In my experience, using only *financial literacy* as the main search term can be too narrow. I conducted a systematic review (see reference below) and had to expand my search terms, as many studies use *financial knowledge*, *financial literacy*, and *financial skills* interchangeably.
- **Overall** – I like the core idea of the paper, but I recommend reworking how you categorize the papers. Try to develop a classification system that is more salient and relevant for researchers. Figure 3 presents some promising topics, but there's noticeable overlap. Consider refining this analysis by using narrower categories to yield more meaningful insights.

**Minor Comments**

- In the abstract, should it be *financial attitudes* instead of *attitude*?
- In the introduction, you mention "young people." It would be helpful to define early on which specific group you are focusing on. When considering young people and financial literacy, possible groups include:
  1. Children (elementary to middle school),
  2. Youth (high school—distinct from K–8 because some U.S. states mandate financial education in high school),
  3. College students, who may overlap with young adults aged 18–24.
    You clarify your focus in the Methods section, but it would be useful to mention this earlier.
- The sentence:
*“Despite the importance of financial education for young people, providing such education has become a problem for many countries, especially for those emerging economies that, in general, do not reflect adequate financial behavior, leading to stagnant growth of the economy (Samy et al., 2008).”*
  contains what seems like an inaccurate generalization. I recommend reworking or removing this sentence.
- **List of references for the 44 studies** – I suggest including this as a separate section from your main reference list, so readers can easily see which studies were included in your systematic review. You’ll likely cite additional literature, so it makes sense to keep them separate (see Blanco et al., 2024, for an example approach).
- The sentence:
*“As shown in Figure 2, the most frequently assessed factor—identified in 26 publications—is financial literacy itself.”*
  isn't a particularly strong insight, given that *financial literacy* was your main search term.
- Similarly,
*“The second most assessed factor is financial knowledge, which is mentioned in 25 papers.”*
  restates your search terms. As noted above, researchers often use *financial knowledge* and *financial literacy* interchangeably.

Xiao, J. J., Huang, J., Goyal, K., and Kumar, S. 2022. “Financial capability: a systematic conceptual review, extension and synthesis.” International Journal of Bank Marketing 40: 1680–1717. https://doi.org/10.1108/IJBM-05-2022-0185

 Lusardi, A. and Mitchell, O. S. 2023. “The importance of financial literacy opening a new field.” Journal of Economic Perspectives 37: 137–154. https://doi.org/10.1257/jep.37.4.137

 Blanco, L. R., Garcia, C., Gulbins, B., & Gutierrez, R. (2024). Systematic review of racial/ethnic and gender differences in financial knowledge in the United States. *Journal of Financial Literacy and Wellbeing*, 1–30. https://doi.org/10.1017/flw.2024.10

Are the rationale for, and objectives of, the Systematic Review clearly stated?

Yes

Is the statistical analysis and its interpretation appropriate?

Partly

If this is a Living Systematic Review, is the ‘living’ method appropriate and is the search schedule clearly defined and justified? (‘Living Systematic Review’ or a variation of this term should be included in the title.)

Partly

Are sufficient details of the methods and analysis provided to allow replication by others?

Partly

Are the conclusions drawn adequately supported by the results presented in the review?

Partly

Reviewer Expertise:

Financial behavior, financial knowledge

I confirm that I have read this submission and believe that I have an appropriate level of expertise to state that I do not consider it to be of an acceptable scientific standard, for reasons outlined above.

# Response

**Published:** 2025-07-02

**Authors:** JHOANY ALEJANDRO VALENCIA ARIAS

We thank the reviewer for their valuable contributions to the rigor of the manuscript. Below, we detail their response point by point:

**Comment**: Contribution of the paper – From reading the abstract, I am not convinced that this paper offers a major contribution. I appreciate the idea behind the study, but I find the structure unconvincing. The terms financial knowledge and behavior are very broad, and the topics suggested for future research are essentially subtopics within these broad categories. I suggest organizing the literature around these various subtopics, then identifying which ones are more commonly addressed and which are underexplored. For example, when focusing on college students, it makes sense to emphasize specific financial behaviors relevant to them—such as student debt and budgeting. In contrast, topics like retirement preparedness and savings may be less applicable to this group. That said, one could argue that introducing college students to concepts like retirement savings and compound interest is crucial. If your paper identifies a gap in that area, it would offer specific insights for stakeholders.

**Response**: We appreciate the reviewer’s valuable observation regarding the need to clarify the specific contribution of our study. In response, we have restructured the literature review and results sections by organizing the broad categories of “financial knowledge” and “financial behavior” into detailed subtopics (e.g., budgeting, student debt, credit card use). This allows for a clearer identification of underexplored areas such as retirement planning, fintech adoption, and financial attitudes in young students. Additionally, the abstract has been revised to better highlight these contributions, and the discussion now includes explicit implications for educational stakeholders and policymakers. We believe these changes clarify the originality and practical relevance of our findings.

**Comment**: Definition of financial literacy – Please elaborate on this definition, as it is central to your study. There are several key papers that discuss this in detail. Given your focus on financial knowledge and behavior, a more comprehensive explanation is necessary. See the references below for possible sources to incorporate.

**Response**: We appreciate the reviewer’s recommendation to strengthen the theoretical foundation of the study by elaborating on the definition of financial literacy. In response, we revised section 1.1 to incorporate several key references

**Comment**: Financial literacy among college students – I recommend expanding this section. The experiences of college students regarding access to financial education programs and their levels of financial knowledge likely vary across regions and countries. This section currently feels weak, especially considering it is the main focus of the paper.

**Response**: The section is strengthened by including a paragraph that expands on the experiences of university students with respect to access to financial education programs and their levels of financial literacy according to regional and national conditions.

**Comment**: Methods section – I suggest reorganizing this section for better flow. For instance, instead of starting with inclusion/exclusion criteria, consider beginning with the search strategy so readers understand your starting point.

**Response**: The methodology section is reorganized for better flow as suggested starting with the inclusion/exclusion criteria and the search strategy so that readers understand the starting point.

**Comment**: Search term – In my experience, using only financial literacy as the main search term can be too narrow. I conducted a systematic review (see reference below) and had to expand my search terms, as many studies use financial knowledge, financial literacy, and financial skills interchangeably.

**Response**: We thank the reviewer for this valuable observation. We recognize that related terms such as “financial knowledge” and “financial skills” are frequently used in the literature and that their inclusion could have broadened the scope of the review. However, expanding the search equation to incorporate these terms would require a full redesign of the study’s methodology and data extraction process, which was beyond the scope of the current article. Nevertheless, we have acknowledged this point as a methodological limitation in the revised version and suggest that future systematic reviews consider a more inclusive keyword strategy to capture a wider range of conceptual approaches.

**Comment**: Overall – I like the core idea of the paper, but I recommend reworking how you categorize the papers. Try to develop a classification system that is more salient and relevant for researchers. Figure 3 presents some promising topics, but there's noticeable overlap. Consider refining this analysis by using narrower categories to yield more meaningful insights.

**Response**: Thank you for this thoughtful and encouraging feedback. In response to your suggestion, we restructured the analysis by developing a more refined and inductively derived classification system. Rather than organizing the results solely under broad categories like “financial knowledge” and “behavior,” we identified six thematic subtopics—such as budgeting, debt management, digital tools, and financial self-efficacy—based on the specific variables reported in each study. This new framework reduces overlap, enhances the salience of the findings, and offers more targeted insights for researchers and stakeholders.

**Comment**: In the abstract, should it be financial attitudes instead of attitude?

**Response**: Corrected as suggested, changed to plural: “Financial Attitudes”, to maintain consistency with the content of the article.

**Comment**: In the introduction, you mention "young people." It would be helpful to define early on which specific group you are focusing on. When considering young people and financial literacy, possible groups include:

 1. Children (elementary to middle school),

 2. Youth (high school—distinct from K–8 because some U.S. states mandate financial education in high school),

 3. College students, who may overlap with young adults aged 18–24.

 You clarify your focus in the Methods section, but it would be useful to mention this earlier.

**Response**: In the introduction section there is a paragraph that clarifies from the beginning the population group defined for the study, which is young university students between 18 and 24 years of age.

**Comment**: The sentence:

 “Despite the importance of financial education for young people, providing such education has become a problem for many countries, especially for those emerging economies that, in general, do not reflect adequate financial behavior, leading to stagnant growth of the economy (Samy et al., 2008).”

 contains what seems like an inaccurate generalization. I recommend reworking or removing this sentence.

**Response**: The paragraph is modified to avoid generalization

**Comment**: List of references for the 44 studies – I suggest including this as a separate section from your main reference list, so readers can easily see which studies were included in your systematic review. You’ll likely cite additional literature, so it makes sense to keep them separate (see Blanco et al., 2024, for an example approach).

 Blanco, L. R., Garcia, C., Gulbins, B., & Gutierrez, R. (2024). Systematic review of racial/ethnic and gender differences in financial knowledge in the United States. Journal of Financial Literacy and Wellbeing, 1–30. https://doi.org/10.1017/flw.2024.10

**Response**: Thank you for this helpful suggestion. Following your recommendation, we have created a separate section at the end of the manuscript that lists the 44 studies included in the systematic review. This distinction makes it easier for readers to identify the primary sources analyzed in the review. Additionally, the article by Blanco et al. (2024), which you kindly recommended, has been incorporated into the main reference list and cited in the theoretical framework.

**Comment**: The sentence:

 “As shown in Figure 2, the most frequently assessed factor—identified in 26 publications—is financial literacy itself.”

 isn't a particularly strong insight, given that financial literacy was your main search term.

**Response**: We agree that the original statement did not offer a meaningful analytical contribution, as it simply reflected the scope of our search strategy. In response, we revised the sentence to emphasize the conceptual ambiguity and varied operationalization of the term “financial literacy” across the included studies.

**Comment**: Similarly,

 “The second most assessed factor is financial knowledge, which is mentioned in 25 papers.”

 restates your search terms. As noted above, researchers often use financial knowledge and financial literacy interchangeably.

**Response**: We recognize that highlighting the frequency of “financial literacy” as a stand-alone outcome did not provide substantial analytic value, especially given the overlap with the main search term. We have revised this phrase to reflect the conceptual ambiguity between “financial literacy” and “financial knowledge” in the literature.

# Reviewer report: Reviewer response for version 1

**DOI:** 10.5256/f1000research.174764.r364298

**Published:** 2025-03-19

**Authors:** Claire Matthews

**Affiliations:** 1 Massey University, Palmerston North, New Zealand

This is an interesting paper, but I have concerns about some elements of it.

 It is unclear what timeframe was used for the literature search.  The timeframe in Figure 3 suggests it was only from 2014, which seems much too short a time frame to do justice to the topic.  Restricting the search to terms in the article title is a major limitation, an issue which is compounded by the search terms use.  I believe the search should have included the abstract.  In addition, I think the search should have included "University" and "College" as separate terms.

 The lack of any reference to the APLUS project at the University of Arizona is likely a reflection of the search limitations.  This is a leading project into financial literacy of university students. The potentially relevant articles include; [Ref 1 and 2]. 

 Research into financial literacy has evolved into financial capability and financial wellbeing - financial literacy has really become an outdated term. The difference between these terms and the evolution of the research away from financial literacy should be discussed in the paper.

 Figure 3 is a useful figure but the timeframes for the various concepts seem very short raising questions about the timeframe used for the research.

 Some attention is needed to the spelling and grammar - in particular there are many unnecessary hyphens throughout the paper.

Are the rationale for, and objectives of, the Systematic Review clearly stated?

Yes

Is the statistical analysis and its interpretation appropriate?

Partly

If this is a Living Systematic Review, is the ‘living’ method appropriate and is the search schedule clearly defined and justified? (‘Living Systematic Review’ or a variation of this term should be included in the title.)

Not applicable

Are sufficient details of the methods and analysis provided to allow replication by others?

Partly

Are the conclusions drawn adequately supported by the results presented in the review?

Yes

Reviewer Expertise:

Financial literacy/capability/wellbeing

I confirm that I have read this submission and believe that I have an appropriate level of expertise to confirm that it is of an acceptable scientific standard, however I have significant reservations, as outlined above.

## References

[1] : Does Financial Satisfaction Vary Depending on the Funding Strategy Used to Pay for College?. *Journal of Family and Economic Issues* .2021;42(3) : 10.1007/s10834-021-09755-7 429-448 10.1007/s10834-021-09755-7

[2] : Earlier financial literacy and later financial behaviour of college students. *International Journal of Consumer Studies* .2014;38(6) : 10.1111/ijcs.12122 593-601 10.1111/ijcs.12122

# Response

**Published:** 2025-04-02

**Authors:** JHOANY ALEJANDRO VALENCIA ARIAS

Dear reviewer, we thank you for your valuable contributions to the research. Below, we respond point by point to your comments:

**Comment**: It is unclear what timeframe was used for the literature search.  The timeframe in Figure 3 suggests it was only from 2014, which seems much too short a time frame to do justice to the topic.  Restricting the search to terms in the article title is a major limitation, an issue which is compounded by the search terms use.  I believe the search should have included the abstract.  In addition, I think the search should have included "University" and "College" as separate terms.

**Response**: The study's time window of analysis (2003-2023) is highlighted. The search equation cannot be modified. These methodological decisions, although necessary to focus the review, may have limited the comprehensiveness of the results. It should also be noted that modifying the search strategy at this point would significantly alter the set of retrieved studies and, consequently, the findings presented in this article. Therefore, it is included in the limitation of the study.

**Comment**: The lack of any reference to the APLUS project at the University of Arizona is likely a reflection of the search limitations.  This is a leading project into financial literacy of university students. The potentially relevant articles include; [Ref 1 and 2]. 

 1. Henager R, Anong S, Serido J, Shim S: Does Financial Satisfaction Vary Depending on the Funding Strategy Used to Pay for College?. Journal of Family and Economic Issues. 2021; 42 (3): 429-448 Publisher Full Text

 2. Xiao J, Ahn S, Serido J, Shim S: Earlier financial literacy and later financial behaviour of college students. International Journal of Consumer Studies. 2014; 38 (6): 593-601 Publisher Full Text

**Response**: Therefore, it is included in the limitation of the study.

**Comment**: Research into financial literacy has evolved into financial capability and financial wellbeing - financial literacy has really become an outdated term. The difference between these terms and the evolution of the research away from financial literacy should be discussed in the paper.

**Response**: A paragraph is added to the discussion acknowledging this evolution of the term. Also, the conceptual differences between the three terms are briefly explained. It justifies why the review focuses on “financial literacy” and how it remains relevant. Finally, it is proposed that future research could take a more integrative approach, including these new concepts.

**Comment**: Figure 3 is a useful figure but the timeframes for the various concepts seem very short raising questions about the timeframe used for the research.

**Response**: It is explained that the time window of the study analysis comprises (2003-2023). However, a study feed is added in response to this observation.

**Comment**: Some attention is needed to the spelling and grammar - in particular there are many unnecessary hyphens throughout the paper.

**Response**: Spelling and grammar errors are corrected. Unnecessary hyphens are eliminated.
